# Effect of Sulindac Sulfide on Metallohydrolases in the Human Colon Cancer Cell Line HT-29

**DOI:** 10.1371/journal.pone.0025725

**Published:** 2011-10-03

**Authors:** Hector Guillen-Ahlers, Jiangning Tan, Francis J. Castellino, Victoria A. Ploplis

**Affiliations:** 1 W. M. Keck Center for Transgene Research, University of Notre Dame, Notre Dame, Indiana, United States of America; 2 Department of Chemistry and Biochemistry, University of Notre Dame, Notre Dame, Indiana, United States of America; 3 Harper Cancer Research Institute, University of Notre Dame, Notre Dame, Indiana, United States of America; Stanford University, United States of America

## Abstract

Matrix metalloproteinase 7 (MMP7), a metallohydrolase involved in the development of several cancers, is downregulated in the *Apc^Min/+^* colon cancer mouse model following sulindac treatment. To determine whether this effect is relevant to the human condition, HT-29 human colon cancer cells were treated with sulindac and its metabolites, and compared to results obtained from *in vivo* mouse studies. The expression of *MMP7* was monitored. The results demonstrated that sulindac sulfide effectively downregulated both *MMP7* expression and activity. Furthermore, activity-based proteomics demonstrated that sulindac sulfide dramatically decreased the activity of leukotriene A4 hydrolase in HT-29 cells as reflected by a decrease in the level of its product, leukotriene B4. This study demonstrates that the effect of sulindac treatment in a mouse model of colon cancer may be relevant to the human counterpart and highlights the effect of sulindac treatment on metallohydrolases.

## Introduction

Non-steroidal anti-inflammatory drugs (NSAIDs), such as sulindac, are chemo-preventive reagents towards colorectal cancers [1], [2], [3]. This effect is consistent with observations *in vitro* in human colon cancer cells [4], as well as *in vivo* in the *Apc^Min/+^* mouse model, where a large decrease in tumor burden is observed [5]. The gastrointestinal complications that develop in NSAID users are well documented [6], and represent an obstacle in the use of these drugs as chemo-preventive agents. The pleiotropic effects of sulindac on colon cancer prevention are still unclear and hinder the development of more specific treatments with diminished side effects. Regarding this issue, it has been shown that sulindac treatment causes changes in extracellular matrix remodeling events, including downregulation of matrix metalloproteinase 7 (*MMP7*) in intestinal adenomas of *Apc^Min/+^* mice [7].

MMP7 belongs to a group of metal ion-dependent proteases. According to the MEROPS database (www.merops.sanger.uk), matrix metalloproteases (MMPs), consisting of 24 members, comprise the M10 subfamily of the MA family of the metallohydrolases clan M. MMPs have been strongly implicated in the development of many cancers. Deletion of *MMP2* and *MMP9* in mice have been shown to reduce pancreatic tumorigenesis by reducing angiogenesis [8]. Pertinent to this study, *MMP7* deletion in *Apc^Min/+^* mice has been shown to strongly reduce intestinal tumor burden [9]. These observations implicate MMP7 as a viable target for the development of novel treatment regimes.

The current study determined the effect of sulindac treatment on MMP7 in the human colon cancer cell line, HT-29, and utilized a global approach to detect altered activities of other metallohydrolases. The results of these investigations are described herein.

## Materials and Methods

### Cell culture

HT-29 cells (American Type Culture Collection, Manassas, VA) were maintained in McCoy's 5A medium (Gibco, Grand Island, NY) with 10% fetal bovine serum at 37°C in an atmosphere of 95% air/5% CO_2_. Sulindac, sulindac sulfide, and sulindac sulfone were purchased from Sigma Aldrich (St. Louis, MO). These drugs were diluted in dimethyl sulfoxide (DMSO). DMSO was added to the media at a final concentration of 1% and cells were cultured in 6-well plates. Once cells were 80% confluent, they were treated with various concentrations of sulindac and its metabolites. After 24 hr, the cells were trypsinized and viable cells counted using the trypan blue method.

### Gene expression

HT-29 cells, at the time of harvest, were trypsinized, centrifuged, and the cell pellet used to extract RNA following the Qiagen protocol. Concentration and quality of the samples were assessed using the spectral profile obtained from the NanoDrop-1000 (Thermo Scientific, Wilmington, DE). Real-time reverse transcription–polymerase chain reaction (RT-PCR) was performed as previously described [7]. Primers and probes designed for human *MMP7*, *MMP25*, *Trypsin1*, and *RPL-19* are shown in Table 1.

**Table 1:** Primers and probes.

Gene^1^		sequence (5′→3′)	T_m_ (°C)	GC (%)
*MMP7*	Forward	GGATGGTAGCAGTCTAGGGATTAACT	61	46
	Reverse	GGAATGTCCCATACCCAAAGAA	61	45
	Probe	CCTGTATGCTGCAACTCATGAACTTGGC	68	50
*RPL-19*	Forward	GCGGATTCTCATGGAACACA	58	50
	Reverse	GGTCAGCCAGGAGCTTCTTG	59	60
	Probe	CCACAAGCTGAAGGCAGACAAGGCC	70	60
*Trypsin1*	Forward	CCCCCTTTGATGATGATGAC	60	50
	Reverse	GATGTCATTGTTCAGAGTCTTC	59	40
*MMP25*	Forward	TCATGAGCTATGCCCTGATG	60	50
	Reverse	AGGGCCTCATAATGGAGTTG	59	50

1 All genes are human.

For RT-PCR studies, total RNA was extracted from HT-29 cells, treated with DMSO or 100 µM sulindac sulfide for 24 hr using the Qiagen protocol. RT-PCR reaction included 2.5× Hotmaster Mix (5 Primer Inc., Gaithersburg, MD), RNase Inhibitor, Multiscribe RT enzyme, passive reference dye ROX, and Sybr Green. Total RNA (25 ng) was revere-transcribed in 30 µl reaction mixture at 48°C for 30 min. The following conditions were used for amplification: pre-denaturation for 5 min at 95°C, 30 cycles of denaturation at 95°C for 20 sec, annealing at 59°C for 20 sec, and extension at 72°C for 25 sec. PCR products were separated by electrophoresis on a 3% agarose gel containing ethidium bromide. A 50 bp DNA ladder (Promega, Madison, WI) was used as the molecular weight marker.

### Western blotting

Proteomes were extracted from HT-29 cells and then treated with sulindac sulfide (100 µM) for 24 hr at 80% confluency. Cells were trypsinized, centrifuged, and then washed in PBS. Cell pellets were then Dounce homogenized and sonicated. The proteome was centrifuged at 100,000× *g* to separate the cytosolic and membrane fractions. The secreted fraction was obtained by centrifugation of the cell media at 100,000× *g*. The proteome was precipitated by the addition of ammonium sulfate. Samples were passed over a PD-10 desalting column (GE Healthcare, Piscataway, NJ) and protein concentrations were determined using the NanoDrop-1000 (Thermo Scientific, Wilmington, DE). Samples were load onto a 10% polyacrylamide gel, electrophoresed, and then transferred to a PVDF membrane. The membranes were incubated overnight at 4°C with polyclonal rabbit-anti-human MMP7 (Abcam, Cambridge MA), and polyclonal rabbit-antihuman active MMP7 (Abcam) at 1 µg/ml in blocking buffer (PBS, 3% nonfat dry milk, 0.1% Tween 20). For MMP25 and α-Tubulin, rabbit anti-human MMP25 (Abcam) and mouse anti-human α-Tubulin (Santa Cruz Technology), respectively, was used. HRP-conjugated anti-rabbit IgG antibody or HRP-conjugated-anti-mouse IgG (Cell Signaling Technology, Boston MA) in the blocking buffer was used as the secondary antibody. Blots were developed using the LumiGLO reagents (Protein Research Products, Gaithersburg, MA) or the Western Lightning ECL Substrate (Perkin Elmer Inc., Waltham, MA).

### Activity-based proteomics for the identification of activated metallohydrolases

Proteomes were adjusted to a concentration of 1 mg/ml in PBS. A cocktail of alkyne probes targeting the active site of metallohydrolases was a generous gift of Benjamin F Cravatt (The Scripps Research Institute, La Jolla, CA). Labeling was performed as previously described [10]. Briefly, probes were added to a final concentration of 1 µM and incubated under UV light for 1 hr. Rhodamine azide was then bound to the probes using click chemistry [11]. Samples were electrophoresed on a 10% polyacrylamide gel and scanned using either a Hitachi FMBio IIe flatbed scanner (MiraBio, Alameda, CA) or the KODAK In-Vivo Multispectral System FX (Carestream Health, Rochester, NY).

### Identification of activity-based labeled proteins

Real-size gel scan prints were used as a template to extract the desired bands and scanned afterwards to assure correct slicing of the gel. Each gel plug was reduced with 10 mM dithiothreitol in ammonium bicarbonate and alkylated with 55 mM iodoacetamide. Samples were digested with 0.1 µg trypsin (Promega, Madison, WI) in 100 µl of 10 mM ammonium bicarbonate and incubated overnight at 37°C. Tryptic peptides were extracted from gel plugs with 50% acetonitrile/49.9% H_2_O/0.1% trifluoroacetic acid followed by 100% acetonitrile and finally with 0.1% trifluoroacetic acid.

Tryptic peptides were chromatographed on a 100 µm×10 cm C18 column (particle size: 5 µm) and eluted using a linear gradient of 3 to 45% acetonitrile in H_2_O over 70 min at room temperature, at a flow rate of 500 nl/min. The eluent was electro-sprayed into the linear quadrupole ion trap (LTQ) mass spectrometer (Thermo-Fisher Scientific, Waltham, MA). Sequest and X!Tandem algorithms were used to search the database utilizing the international protein index (IPI) mouse database.

### Leukotriene B4 enzyme immunoassay

At 80% confluence, HT-29 cells were incubated with either sulindac sulfide or DMSO for 24 hr. The secreted proteome was obtained and concentrated by vacuum centrifugation. Proteomes were adjusted to 5 mg/ml and LTB4 quantified with an enzymatic immunoassay kit (Cayman Chemical, Ann Arbor, MI).

## Results

### The reduced metabolite of sulindac, sulindac sulfide, selectively increases cell death of HT-29 cells

**Figure 1:**
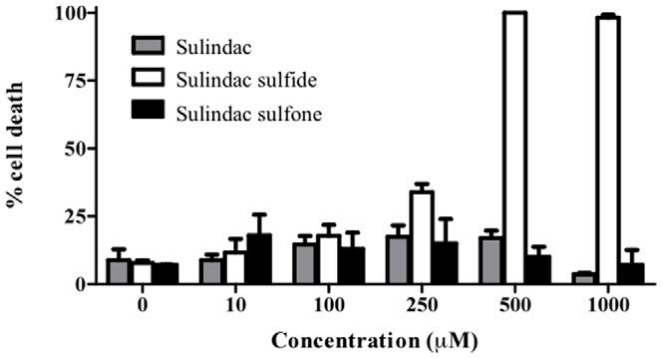
Percent cell death of HT-29 human colon cancer cells. At 80% confluency, cells were treated with increasing concentrations of sulindac (grey), sulindac sulfide (white), and sulindac sulfone (black).

HT-29 cells were treated with sulindac, sulindac sulfide, and sulindac sulfone at concentrations ranging from 10 to 1000 µM (Figure 1). Starting at 250 µM, sulindac sulfide significantly increased cell death, reaching 100% cell death at 500 µM. In contrast, sulindac and sulindac sulfone failed to significantly increase cell death, even when the drugs where added at mM concentrations.

### Sulindac sulfide reduces *MMP7* at the mRNA, protein, and activity levels

**Figure 2:**
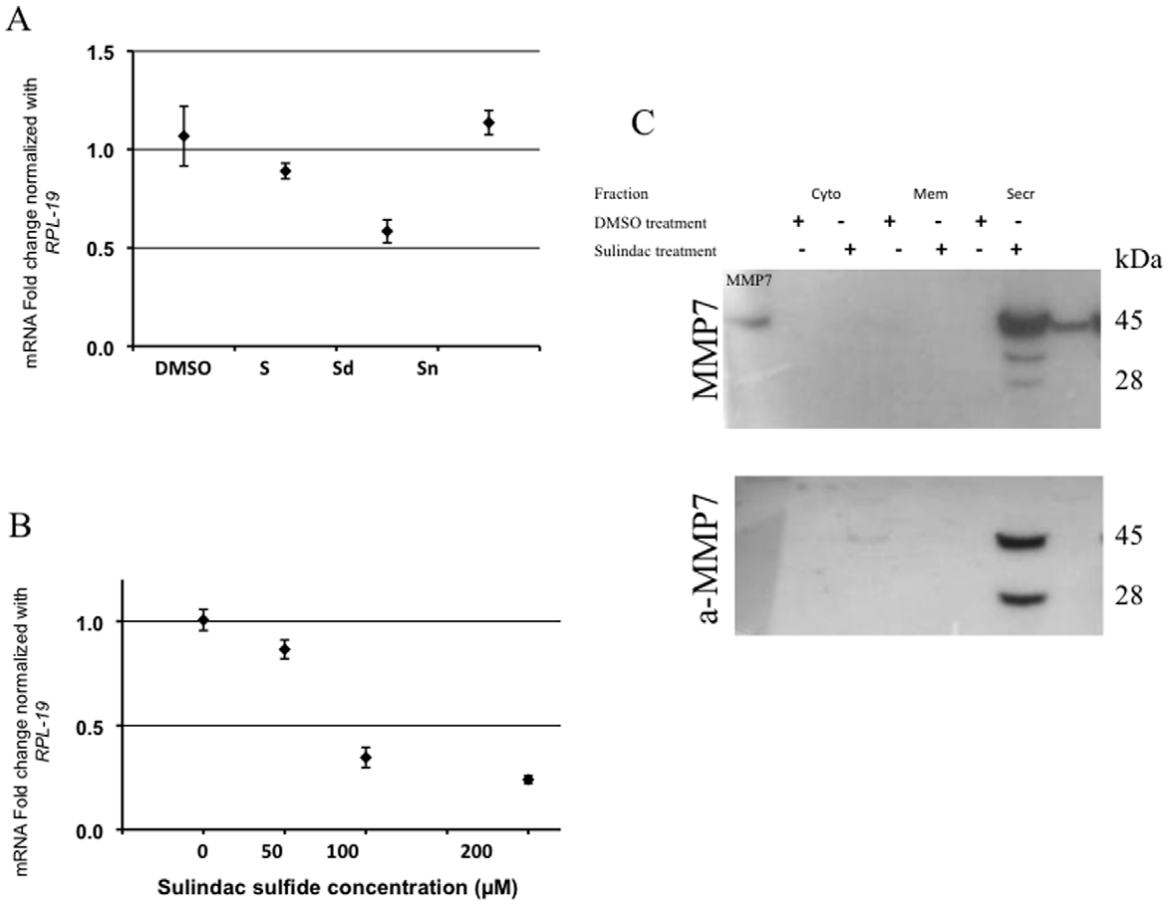
MMP7 expression in sulindac-treated cells. (**A**) Real time RT-PCR of RNA from HT-29 cells after 24 hours of treatment with DMSO, sulindac (S), sulindac sulfide (Sd) and sulindac sulfone (Sn). The results were obtained using the ΔΔCt method with *hRPL-19* as the housekeeping gene. C_T_ values between 20.10 and 20.56 across all samples show constant levels of *hRPL-19* expression. (**B**) The expression of *hMMP7* after 24 hours of sulindac treatment. The downregulation of *hMMP7* was significant at all concentrations. The results were obtained using the ΔΔCt method with *hRPL-19* as the housekeeping gene. C_T_ values between 23.22 and 23.56 across all samples show constant levels of *hRPL-19* expression. (**C**) Cytosolic, membrane, and secreted proteome fractions extracted from DMSO- and sulindac sulfide-treated HT-29 cells were subjected to western blot analysis using an antibody which does not differentiate between active and inactive MMP7 (top), and an antibody that only recognizes the active site (bottom). The expected band for MMP7 appears at 28 kDa, however a band at 45 kDa is often reported, possibly a dimer. Cyto denotes cytosolic fraction; Mem, membrane fraction, Secr, secreted fraction, and aMMP7, active MMP7.

It was reported that two days of sulindac treatment is sufficient to downregulate MMP7 in tumors of *Apc^Min/+^* mice [7]. In order to determine which metabolite effectively diminished MMP7, HT-29 cells were treated with sulindac and its metabolites at 100 µM, a concentration not toxic to the cells as demonstrated in figure 1, for 24 hr. The expression profile of *MMP7* was compared to the results previously obtained in mouse studies (Figure 2A). Only the active metabolite, sulindac sulfide, was capable of significantly reducing the expression of *MMP7.* A titration curve using different concentrations of sulindac sulfide revealed that even at 50 µM, *MMP7* showed a small (14%), but statistically significant downregulation of expression (p = 0.044) (Figure 2B). At 100 µM, a decrease of 65% was observed (p = 0.000001). The difference between 100 µM and 200 µM was less dramatic (11%), but was still significantly different between these two concentrations (p = 0.017).

Western blotting of MMP7, as well as the active site of MMP7, was performed on cytosolic, membrane, and secreted fractions of HT-29 proteomes after 24 hr of sulindac sulfide treatment at 100 µM (Figure 2C). In both cases, MMP7 was only detected in the secreted fractions of the proteome. Sulindac sulfide treatment showed a clear reduction of MMP7 in the secreted fraction at a 100 µM concentration. Western blot analysis targeting the active site of MMP7 revealed a complete disappearance of detectable active MMP7 after sulindac sulfide treatment.

**Figure 3:**
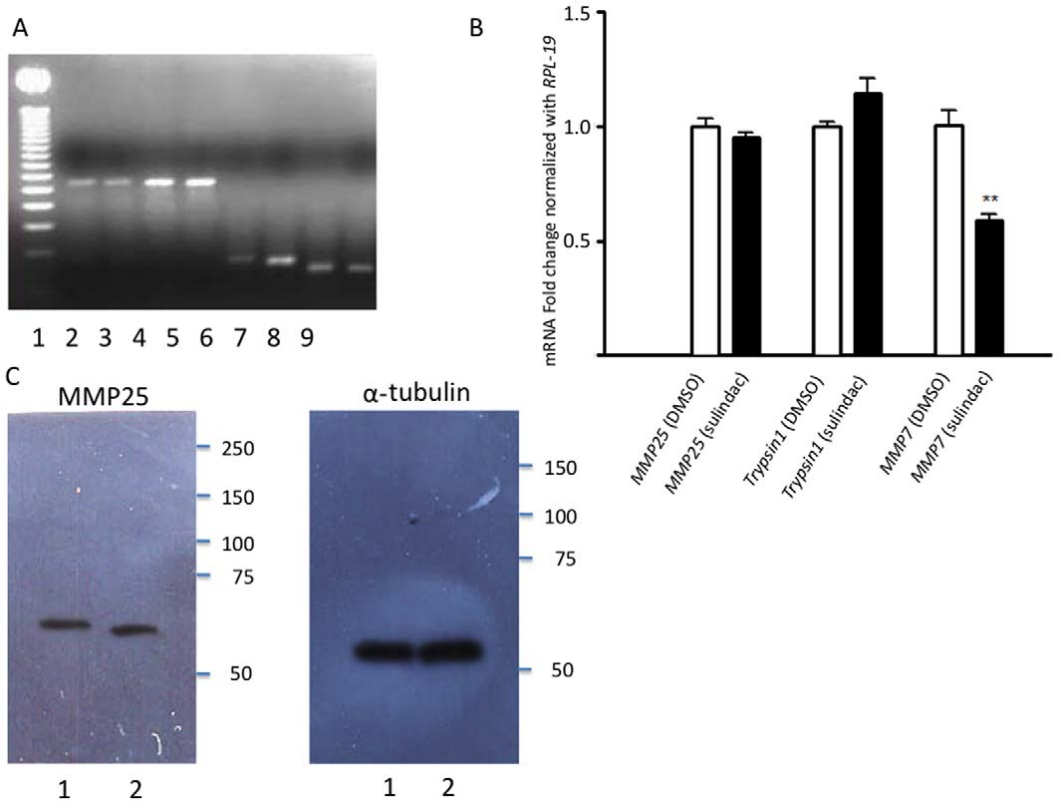
MMP7, MMP25, Trypsin1 and RPL-19 expression in sulindac sulfide-treated cells. (**A**) PCR product of RT-PCR of RNA from HT-29 cells after 24 hours of treatment with DMSO or sulindac sulfide. Lane 1, 50 bp DNA ladder; lane 2, *MMP25* after sulindac sulfide treatment; lane 3, *MMP25* after DMSO treatment; lane 4, *Trypsin1* after sulindac sulfide treatment; lane 5, *Trypsin1* after DMSO treatment; lane 6, *MMP7* after sulindac sulfide treatment; lane 7, *MMP7* after DMSO treatment; lane 8 *RPL-19* after sulindac sulfide treatment; lane 9, *RPL-19* after DMSO treatment. Results demonstrate that only *MMP7* expression is affected by sulindac sulfide treatment. (**B**) Quantitative RT-PCR of *MMP25*, *Trypsin1*, and *MMP7* after DMSO or sulindac sulfide treatment. The results were obtained using the ΔΔCt method with *hRPL-19* as the housekeeping gene. Results indicate quantitative differences in *MMP7* expression after sulindac sulfide treatment relative to DMSO treatment. (**C**) Western blot of secreted fraction of HT-29 cells demonstrating that the protein levels of MMP25 in the membrane fraction are similar between sulindac sulfide and DMSO treated cells.

In order to determine if other MMPs, other classes of proteases, and the housekeeping gene, RPL-19, were affected by sulindac in HT-29 cells, RT-PCR (whole cell extracts) and Western blot (secreted, cytosolic, and fractions) analyses were performed. mRNA for MMP25, trypsin1, and RPL-19 were not altered after sulindac sulfide treatment (Figure 3A, B). Western blot analysis of the membrane fraction of these cells confirmed RT-PCR results for MMP25 (Figure 3C).

### Activity-based protein profiling targeting metallohydrolases reveals a decrease in LTA4H activity

**Figure 4:**
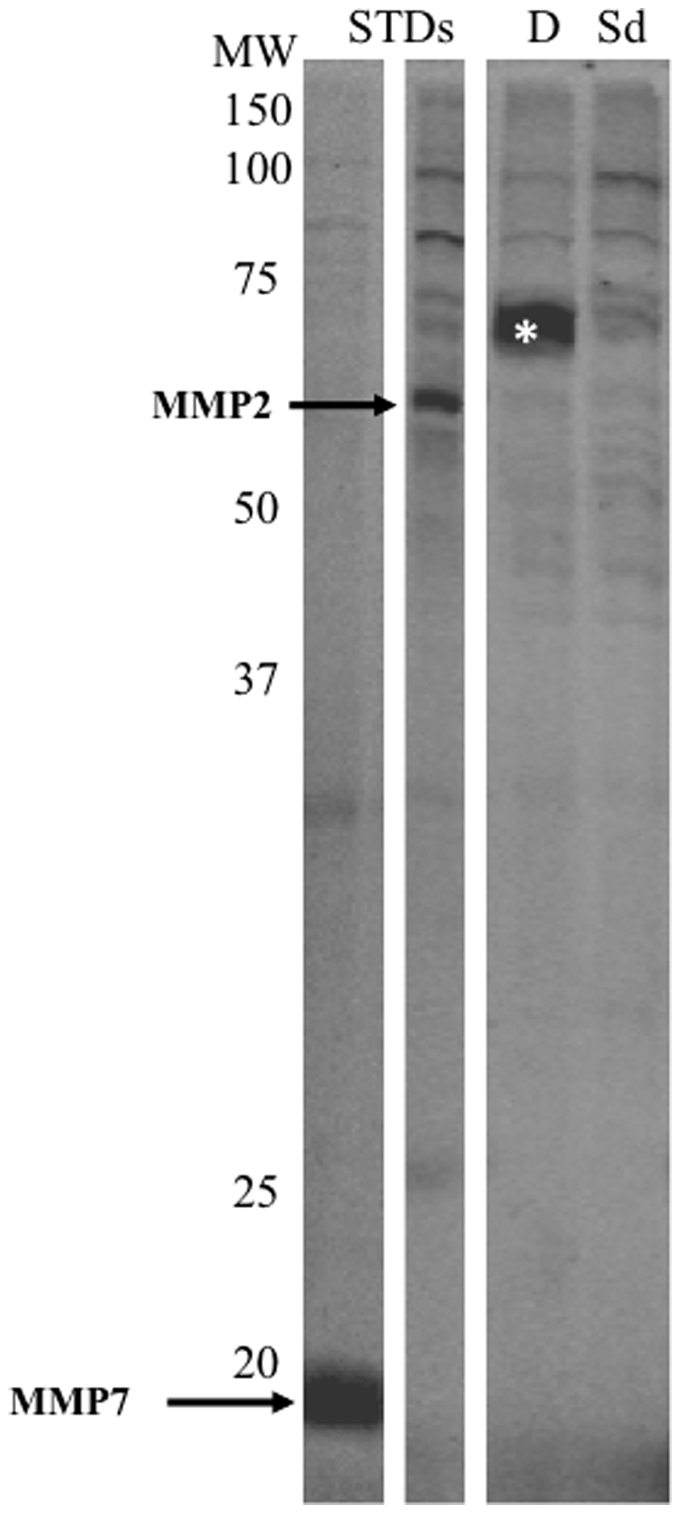
Activity based proteomics and LTA4H in HT-29 cells. Metallohydrolase activity-based labeling of HT-29 cells secreted proteomes. MMP2 and MMP7 standards were added to cell proteomes (arrows). The band displaying a strong decrease (*) after sulindac sulfide treatment was extracted and analyzed by mass spectrometry. MS/MS spectra of LVVDLTDIDPDVAYSSVPYEK and LTYTAEVSVPK peptides corresponded to amino acids 366–386 and 155–165, respectively, of the LTA4H protein. Protein content across all samples was adjusted to 1 mg/ml and the equivalent of 15 µg of proteome was added per lane. MW denotes molecular weight; STDs, standards, Sd, sulindac sulfide-treated and D, DMSO-treated.

Proteomes extracted from HT-29 cells after 24 hr of treatment with sulindac sulfide or DMSO were labeled with a probe cocktail targeting the metallohydrolase superfamily and run on a 1D gel. A strong band between 60 and 65 kDa was observed in HT-29 cells treated with DMSO. Sulindac sulfide-treated cells showed a faint band of similar molecular weight. The bands were excised and analyzed by mass spectrometry. The samples were run by linear quadrupole MS/MS (Figure 4) and the results matched with the MEROPS database for metallohydrolases. There were two peptides (LTYTAEVSVPK and LVVDLTDIDPDVAYSSVPYEK) detected in the ∼70 kDa band that matched leukotriene A4 hydrolase (LTA4H). The predicted molecular weight of LTA4H is 69 kDa. LTA4H has previously been shown to be a target for this metalloprotease probe cocktail (10).

**Figure 5:**
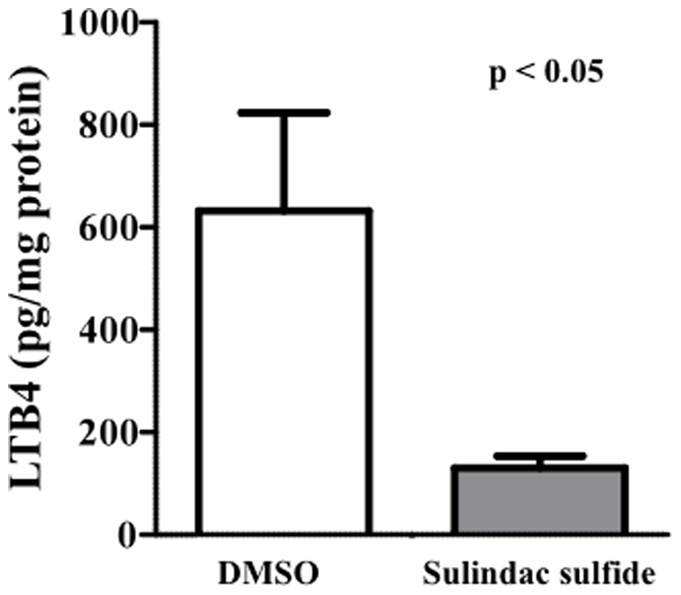
Enzymatic immunoassay for LTB4. Secreted proteomes of DMSO-treated and sulindac sulfide-treated cells were analyzed for LTB4 levels using an enzymatic immunoassay.

In order to validate the reduction in LTA4H activity, an enzymatic immunoassay was used to measure leukotriene B4 (LTB4) levels after sulindac treatment (Figure 5). Sulindac sulfide-treated cells showed strongly reduced levels of LTB4 when compared to DMSO-treated cells.

## Discussion

The present study demonstrated that sulindac sulfide is capable of reducing the RNA and protein levels of MMP7, which is consistent with what was observed in mouse studies [7]. Through activity-based protein profiling, it was demonstrated that sulindac sulfide dramatically reduces the activity of LTA4H. This result was validated by decreased levels of LTB4.

The involvement of metalloproteases in colon cancer has previously been identified but the development of treatment strategies targeting these enzymes has been unsuccessful [12]. One problem is the low specificity of metalloprotease inhibitors that have made it to clinical trials. MMP7, however, has consistently been found to be relevant in colon cancer and its animal models [9], [13]. The present study indicated that sulindac sulfide is able to reduce the expression of *MMP7* in the human colon cancer cell line HT-29 which was also observed, *in vivo*, in *Apc^Min/+^* mice [7]. In order to determine if other MMPs or other classes of proteases were also altered, MMP25, a membrane-associated MMP that is highly expressed in cancer cells [14], and trypsin1 were analysed and shown to be unaffected by sulindac sulfide treatment. Additionally, the housekeeping gene RPL-19 was also not affected by this NSAID. Nobiletin, another agent with anticancer properties, has also been shown to downregulate *MMP7* [15] in HT-29 cells. Several mechanisms have been proposed by which MMP7 promotes tumor growth. Several of those studies link MMP7 with a disrupted Fas-mediated apoptotic response [16], [17], [18], and inflammation-related facilitation of tumor growth has been proposed to be caused by MMP7 cleavage of Fas ligand [19].

Zinc metallopeptidases consist of 12 members and belong to the family of metallohydrolases. These proteases have been implicated in several cancers including aminopeptidase N in colon cancer [20], cystinyl aminopeptidase in renal cancer [21], and LTA4H in lung and colon adenocarcinomas [22]. This is the first study linking sulindac to regulating LTA4H. The downregulation of activity of LTA4H, and decrease in tumor burden, after sulindac treatment supports studies in which it has been shown to be upregulated in colon cancer. Furthermore, [6]-gingerol, a natural component with antitumorigenic properties, has been found to suppress cancer growth by LTA4H inhibition [23]. The decrease in LTA4H activity was validated by the observation that lower LTB4 levels were found after sulindac treatment. LTB4 is a well known eicosanoid with chemotactic properties and has been shown to stimulate the proliferation, *in vitro*, of HT-29 colon cancer cells [24].

There has been no other report of the involvement of sulindac, or any other NSAID, on MMP7 or LTA4H expression. However, in preliminary studies, we have shown that sulindac is not the only NSAID capable of downregulating *MMP7*, i.e., aspirin (data not shown), which suggests that *MMP7* is downregulated by a shared NSAID mechanism [7], [25] and not a unique property of sulindac.

In summary, the present study identified two candidates, previously reported to be highly relevant in tumor development, that are altered after sulindac treatment. New treatment modalities selectively targeting MMP7 and LTA4H, individually or in combination, offer new therapeutic approaches that take advantage of the benefits of sulindac treatment, but potentially without the adverse side effects of this drug.
